# Is Virulence Gene *papGII* a Predictor of Urosepsis in Uropathogenic *E. coli*?

**DOI:** 10.3390/idr18040063

**Published:** 2026-06-24

**Authors:** Nihitta Hanna, Suji Thangamani, Rosemol Varghese, Jiji Smila Arockiasamy, Balaji Veeraraghavan, Rani Diana Sahni

**Affiliations:** 1 Christian Medical College, Vellore 632004, India; 2 Department of Clinical Microbiology, Christian Medical College Hospital, Vellore 632004, India

**Keywords:** UPEC, *E. coli*, *papGII*, urosepsis, UTI, pyelonephritis

## Abstract

Background: Urosepsis is a life-threatening condition accounting for approximately 20–30% of all sepsis cases and typically arises from ascending infection by uropathogenic *Escherichia coli* (UPEC). Disease progression is mediated by virulence factors, including adhesins, iron acquisition systems, and toxins. Among these, P fimbriae, particularly *papGII* adhesin subunit, have been implicated in the transition from uncomplicated urinary tract infection (UTI) to severe urosepsis. This study aimed to evaluate whether *papGII* carriage, alone or in combination with other UPEC virulence determinants and clinical risk factors, can predict urosepsis. Methods: A total of 60 paired *Escherichia coli* isolates from concurrent blood and urine samples of adults with clinical sepsis were collected between January and June 2024. Control isolates were obtained from patients with cystitis (n = 28) and pyelonephritis (n = 32). Polymerase chain reaction (PCR) assays were used to detect fifteen virulence-associated genes, including the *pap* operon (with *papG* allelic variants), the type 1 fimbriae (*fimH)*, S fimbriae (*sfaS*), curli fimbriae (*csgA*), *afa/Dr* adhesin operon genes, cytotoxic necrotizing factor 1 (*cnf1*), and the aerobactin biosynthesis (*iucD*) and receptor (*iutA*) genes. Associations between gene carriage and clinical groups were analyzed using chi-square tests. Results: The incidence of urosepsis increased with age, peaking in the 60–69-year age group. Renal disease and catheterization were identified as significant risk factors (*p* < 0.05). More than 95% of UPEC isolates carried the *csgA* gene associated with biofilm formation and the *iucD* gene. The α- hemolysin toxin (*hlyA*) was significantly associated with urosepsis [X^2^(1, N = 120) = 6.62, *p* = 0.03]. No significant differences were observed in the carriage of *papA*, *papC*, or *fimH*. Although *papGII* was present in 65% of urosepsis-associated UPEC isolates, it did not demonstrate a statistically significant independent association with urosepsis [*p* = 0.1]. Conclusion: This study demonstrates that while *papGII* may contribute to the pathogenic potential of UPEC and facilitate systemic infection, it is not a reliable independent predictor of urosepsis.

## 1. Introduction

Urosepsis is a complex life-threatening syndrome caused by an inflammatory response to a systemic infection originating in the urogenital tract [1]. It accounts for approximately 25% of all sepsis cases in adults and is associated with a mortality rate of 20–40%, underscoring the need for early diagnosis and timely management [2]. Gram-negative coliforms are the predominant causative agents of urinary tract infections (UTIs), with *Escherichia coli* (*E. coli*) responsible for nearly half of all cases. Other commonly implicated organisms include *Klebsiella* spp., *Enterococcus* spp., *Proteus* spp., and *Pseudomonas aeruginosa* [1].

UTIs most commonly arise from an ascending infection following perineal colonization by intestinal or skin microbiota. Alternatively, direct inoculation of the urinary tract may occur during catheterization, particularly in the setting of suboptimal aseptic techniques [3]. The pathogenic potential of uropathogenic *E. coli* (UPEC) is largely attributed to the acquisition of virulence genes through horizontal gene transfer, mediated by mobile genetic elements such as pathogenicity islands, transposons, bacteriophages, and plasmids [4,5]. A key step in the pathogenesis of urosepsis is the ability of UPEC to adhere and colonize the uroepithelium. This is facilitated by fimbrial and afimbrial adhesins [6,7]. Additional virulence mechanisms include iron acquisition via siderophores and heme receptors, as well as the production of toxins, such as α-hemolysin and cytotoxic necrolyzing factor 1, and serum resistance ability, which contribute to immune evasion and tissue damage [6,7]. P fimbriae are extensively studied virulence factors in UPEC and are strongly implicated in invasive diseases [7]. P fimbriae are encoded by a pyelonephritis-associated pilus (*pap*) operon, consisting of subunit proteins *papC*, *papA*, *papK*, *papE*, *papF*, and *papG.* The *papG* adhesin, located at the tip of the P fimbriae, mediates bacterial attachment to uroepithelial cells. Four allelic variants of *papG* (I–IV) have been described. Among these, *papGI* is rarely detected in UTIs, while *papGIV* remains insufficiently characterized. In contrast, *papGII* is frequently identified in UPEC isolates associated with urosepsis, with reported prevalence rates of 64–72% [8,9,10]. This adhesin preferentially binds to Gb04 glycosphingolipid receptors on the uroepithelial cells and has been linked to progression from lower UTIs to acute pyelonephritis and urosepsis. The human kidney cells express both globo-series glycosphingolipid Gb04 and Gb03 receptors, with Gb03 present in more abundance than Gb04. In contrast, bladder epithelial cells express Gb04 and Gb05 receptors, with Gb04 again less abundant. While both *papGII* and *papGIII* bind to Gb04 receptors, *papGIII* additionally has the ability to bind to Gb05 receptors. This results in a strong association of *papGII* with pyelonephritis and bacteremia, and *papGIII* with cystitis. In 2023, a genome-wide study (bGWAS) identified *papGII* as a key genetic determinant in the progression of UTIs to urosepsis, suggesting a potential role as a diagnostic or prognostic biomarker [11,12,13,14].

In this context, the present study aimed to evaluate the role of virulence gene carriage, particularly *papGII*, along with associated clinical risk factors, in the progression of *E. coli* UTIs to urosepsis. This study further explores whether *papGII* alone may serve as a predictor of urosepsis.

## 2. Methods

### 2.1. Study Design and Setting

This is a cross-sectional observational study conducted in the Department of Clinical Microbiology, Christian Medical College Hospital, Vellore, India.

### 2.2. Study Population and Isolate Selection

The inclusion criteria for the urosepsis group included *E. coli* isolated from blood cultures collected within 7 days before or after a urine culture demonstrating significant bacteriuria (>10^5^ CFU/mL) with *E. coli* of an identical antibiogram in symptomatic patients [9]. These paired *E. coli* isolates from both the blood and midstream clean-catch urine samples were collected between January 2024 and June 2024, and stored in nutrient agar deeps until further testing.

An equal number of *E. coli* isolated in urine specimens from patients diagnosed with clinical cystitis and pyelonephritis during the same study period were included as controls for comparison of virulence gene carriage and associated risk factors.

All *E. coli* were identified by conventional quality-passed biochemicals and MALDI-TOF VITEK^®^ MS, bioMérieux, Marcy-l’Étoile, France, Version 3.2.0.

### 2.3. Bacterial Processing and DNA Extraction

All isolates were sub-cultured onto quality-passed MacConkey agar plates to ensure purity. Genomic DNA was extracted from pure bacterial growth. A loopful of bacterial growth was suspended in 200 μL of normal saline, and DNA extraction was performed using the QIAmp DNA Mini Kit (Qiagen, Hilden, Germany) according to the manufacturer’s instructions. DNA was eluted in 100 μL of elution buffer and stored at −20 °C until use.

### 2.4. PCR Amplification of Virulence Genes

Conventional polymerase chain reaction (PCR) was performed using previously validated primers to detect *papGII* and other virulence-associated genes, including those encoding adhesins, toxins, and siderophores. Adhesin genes included P fimbriae—*papGI*, *II*, *III*, *IVa*, *papA*, and *papC* genes, type 1 fimbriae (*fimH*), afimbrial adhesin (*afaC*), S fimbriae—(*sfaS*), and curli fimbriae—(*csgA*); toxin genes included *hlyA* (α-hemolysin) and *cnf1*, while aerobactin synthesis genes included *iutA* and *iucD* [15,16,17].

PCR controls were included in each run. *E. coli* ATCC 35218 was used as a positive control strain for *papA*, *papC*, *cnf1*, *sfaS*, *hlyA*, *csgA*, and *papGIII,* while *E. coli* ATCC 25922 was used as a control for *fimH*, *iutA/iucD*, and *papGII*. In-house DNA controls were used for the detection of other targets.

PCR cycling conditions for *papGI–IVa* primers included an initial denaturation at 95 °C for 5 min, followed by cycles of denaturation at 94 °C for 60 s, annealing at 60 °C for 90 s, and extension at 72 °C for 90 s, with a final extension at 72 °C for 10 min, and a hold at 4 °C.

The PCR products were analyzed by electrophoresis on 2% agarose gels with a 100 bp DNA ladder to confirm expected amplicon sizes.

### 2.5. Clinical Data Collection

Clinical data was retrieved from the electronic medical records, including age, gender, residence, presenting symptoms, and comorbidities (diabetes mellitus, hypertension, malignancy, and renal disease), as well as a prior history of UTI, catheterization, and urinary calculi.

### 2.6. Statistical Analysis

Associations between virulence gene carriage (chiefly *pap* genes) and clinical risk factors were evaluated using the chi-square test. A *p*-value < 0.05 was considered statistically significant.

## 3. Results

Sixty paired isolates of *E. coli* from the urine and blood cultures submitted within one week of each other from 60 patients with clinical urosepsis were included in this study. Concomitantly, 32 *E. coli* from patients with pyelonephritis and 28 isolates from cystitis cases were included. The patient demographics and underlying risk factors were compared in the three clinical levels of infection.

### 3.1. Demographic Results

**Figure 1:**
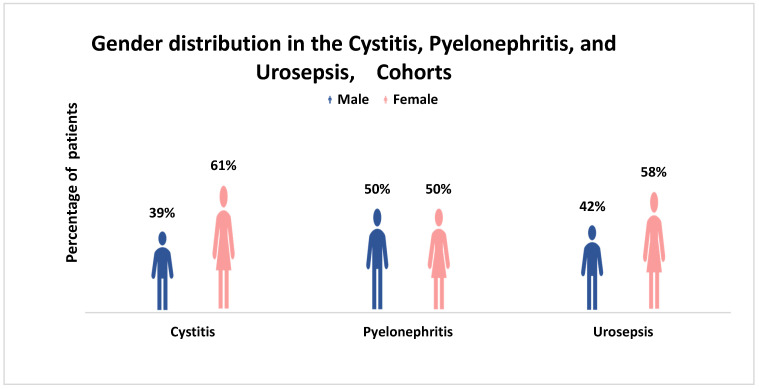
Cis-Gender Distribution of UPEC-Isolated Patients Across Different Levels of Urinary Tract Infection at CMC, Vellore (January–June 2024).

A female predominance was noted in the acquisition of both lower tract (61%) and invasive infections (58%) (Figure 1).

**Figure 2:**
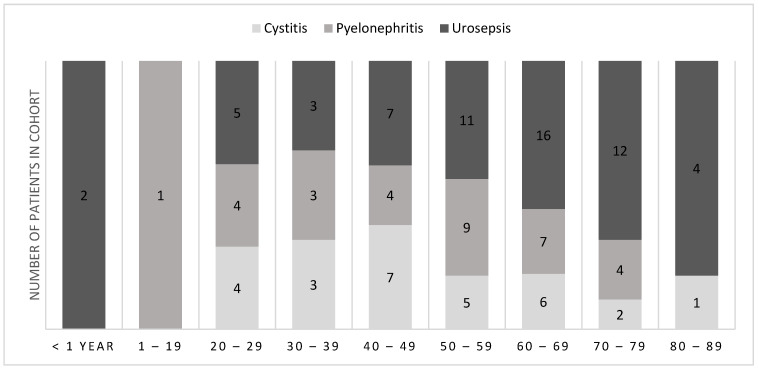
Age Distribution of UPEC-Isolated Patients Across Different Levels of Urinary Tract Infection at CMC, Vellore (January–June 2024).

Clinical urosepsis was noted from the neonatal period to the elderly, steadily increasing with advancing age, with the peak occurrence in the 60–69 age group. Pyelonephritis was seen in the second decade and peaked in the sixth decade, while cystitis was seen among the 20–80 age group, peaking in the fifth decade (Figure 2).

Underlying risk factors contribute to the ascension of organisms and the development of urinary tract infections.

A chi-square test of independence was performed to examine the causal relation between risk factors and urosepsis. The presence of a catheter and the underlying renal disease were significant risk factors for the development of urosepsis (Table 1).

### 3.2. Molecular Characterization of UPEC Virulence Genes by PCR

The adhesion-associated genes: *papGII* carriage progressively increased from the lower (42.9%) to upper tract (56.3%) to the isolates associated with urosepsis (66.7%). No amplifications were observed for *papGI* [17]. The *pap* operon *papA* and *papC* also increased with ascending infection and were found in 68.3% and 71.7% among urine isolates associated with urosepsis (Table 2).

The type 1 fimbriae adhesin gene *fimH*, with receptors throughout the tract, was found in 67.9% of cystitis-associated infections, 81.3% of pyelonephritis, and 85.0% of urosepsis. The core virulence factor essential for biofilm formation, curli fimbriae subunit *csgA*, was detected in almost all isolates, 98.7% of urine and 95% of blood urosepsis isolates, while 100% of isolates causing cystitis also carried the gene. Among the urosepsis strains, 66.6% (n = 40/60) possessed *papGII*, while 28.3% (n = 17/60) were *papGII*-negative isolates. (Table 3). Both the *papGII* and the *papGII*-negative strains isolated from urosepsis had a larger number and a similar type of virulence factors profile, such as *iucD*, *iutA*, *csgA*, *and Fim H* (Table 4), than those associated with cystitis and pyelonephritis.

Toxin genes: The α-hemolysin gene *hlyA* was identified in 48.3% of urine compared to 40.0% of blood isolates. A chi-square test of independence performed to examine the relation between *hlyA* and urosepsis, *X*^2^(1, n = 120) = 6.62, *p* = 0.03, was significant.

Iron uptake gene: survival essential genes, the aerobactin receptor *iutA,* and biosynthesis *iucD* gene were identified in all levels of infection, 81.7% from urine and blood isolates, 85.7% in lower tract infections, and highest in the upper tract, 90.6%.

## 4. Discussion

Uropathogenic *E. coli* express a wide range of virulence factors. Among these, P fimbriae encoded by the *pap* A-K operon are considered key determinants associated with ascending infection to pyelonephritis and urosepsis [10,18]. The adhesin *papG* located at the pilus tip mediates bacterial attachment to glycan receptors of the globo-series glycosphingolipids, including the Forssman antigen (Gb5), globotetraosylceramide (Gb4), and globotriaosylceramide (Gb3) [19]. Notably, the *papGII* allele has been found to be associated with 65% of bacteremia/urosepsis in bacterial genome-wide association studies (GWAS) [9,14].

In the present study, 65% of the UPEC isolates associated with urosepsis carried the *papGII* gene; however, this association was not statistically significant. These isolates displayed a higher virulence gene content, which is consistent with previous studies [20,21]. Owrangi et al. reported an increased adhesion and invasion among the urosepsis and hospital-acquired UTI isolates [21]. More than 90% of the isolates co-harbored *papA*, *papC*, *iutA*, *iucD*, and *csgA* compared with the *papG*-negative strains (Table 4). Further, more than 95% of *papGII*-positive strains carried *papA* and *papC* genes, reflecting the intact nature of the *pap* gene operon in these strains in contrast to *papGIII* or *papG*-negative isolates [20]. Importantly, 37% of *papGII*-negative isolates are also associated with urosepsis (Table 3), indicating that *papGII* carriage alone does not predict progression to urosepsis.

The type 1 fimbriae adhesin gene *fimH* binds to receptors throughout the urinary tract, including the desmoglein-2, on renal epithelial cells [22]. This interaction facilitates initial attachment, colonization, biofilm formation, and rapid bacterial replication, chiefly in the lower tract, while also contributing to ascending infections [15,16]. During early infection, *fimH*-dependent invasion of bladder epithelial cells enables formation of intracellular bacterial communities (IBCs), promoting bacterial persistence, immune evasion, and recurrent urinary tract infections [23].

Curli fimbriae encoded by *csgA* complements type 1 fimbriae *(fimH)* by promoting robust adherence and biofilm development. Curli fimbriae-associated biofilms improve UPEC survival by providing protection against host immune defenses, including cationic antimicrobial peptides. Consistent with previous studies, *csgA*-expressing strains demonstrate increased colonization efficiency and tissue damage in both the bladder and kidneys [24]. Although *fimH* and *csgA* are individually considered weaker biofilm contributors compared to *sfa* [25], their co-occurrence in 80% of the isolates suggests a synergistic role in colonization and persistence. Notably, among the *papGII*-positive isolates, 84% carried *fimH,* and 97% carried *csgA*, and among *papG*-negative isolates, 80.4% and 95.6% carried *fimH* and *csgA,* respectively. The comparable prevalence of these adhesins in both groups suggests that *fimH* and *csgA* may act as independent contributors to infection, persistence, and ascending spread, irrespective of *papGII* carriage.

The pore-forming toxin α-hemolysin (*hlyA*) has been consistently implicated in the UPEC pathogenicity, contributing to epithelial damage, inflammation, and renal injury [26]. In this study, *hlyA* showed a significant association with urosepsis (*p* = 0.03) and was present in 31.3% of pyelonephritis isolates, supporting its role as a marker of disease severity. These findings are consistent with prior reports linking *hlyA* expression to adverse clinical outcomes and progression to systemic disease [27].

The iron acquisition genes *iutA* and *iucD* co-existed in 84% of the total isolates, with 56.6% observed among *papGII*-positive strains and 27.5% among *papG*-negative strains. Their presence reflects adaptation to iron-limited host environments through carriage of *iutA* and *iucD*, siderophore-mediated uptake (aerobactin system) [9]. The widespread distribution of these genes across urosepsis, cystitis, and pyelonephritis groups likely reflects their co-localization on ColV virulence plasmids [28].

Overall, the presence of type 1 and type II adhesin gene carriage, combined with efficient iron acquisition systems and biofilm-forming capacity, highlights the invasive and persistence potential of UPEC across the spectrum of urinary tract infections. These findings suggest that progression to urosepsis is driven by a multifactorial interplay of virulence determinants, with individual strains exhibiting a distinct virulence gene profile [29].

In addition to bacterial factors, host susceptibility plays a critical role in determining infection outcomes [30,31,32]. Even strains lacking classical virulence determinants such as *papGII* may cause invasive disease in susceptible individuals, highlighting the interplay between host defenses and bacterial potential [10,33,34]. In the present study, the cis-female gender, an underlying renal disease, and catheterization were identified as significant risk factors for urosepsis (*p* < 0.05). This contrasts with previous reports by Prabhu et al. [17] and Bijou et al. [35], which identified diabetes mellitus as the primary risk factor; however, these studies focused on cases of urosepsis. In our cohort, although diabetes (55%) and renal disease (53.3%) were common, neither showed a statistically significant difference compared to cystitis cases. These observations reinforce the importance of host-related factors in UTI classification and progression [36].

In summary, the findings of this study suggest that while *papGII* may enhance the likelihood of systemic infection, it is not an independent predictor. Rather, progression to urosepsis appears to result from a complex interplay between bacterial virulence traits and host factors, including gender, renal disease, and indwelling medical devices. The bacterial genome-wide association studies (GWAS) have identified genetic variants linked to disease risk, which represent correlations, but further integrative approaches combining genomic, functional, and clinical data are required to better elucidate the determinants of UPEC pathogenicity and disease progression [37,38].

### Study Limitation

The study limitation includes a smaller number of samples in both groups and the selection of 15 key virulence genes among the UPEC virulence factors.

## 5. Conclusions

*papGII* is enriched within a more virulent genetic background and is not an independent predictor of urosepsis. Future research must integrate genomic, functional, and clinical data while accounting for host comorbidities to better understand the biological mechanisms underlying urosepsis.

## Figures and Tables

**Table 1:** Risk Factors for Urosepsis, Cystitis, and Pyelonephritis.

Risk Factors	Urosepsis (n = 60)	Cystitis (n = 28)	Pyelonephritis (n = 32)	Chi-Square *X*^2^	*p*-Value *
Diabetes mellitus	55.0%	42.9%	43.8%	1.6	0.44
Hypertension	40.0%	53.6%	37.5%	2.4	0.3
Malignancy	13.3%	7.1%	9.4%	0.85	0.7
Previous History of UTI	8.3%	7.1%	21.9%	4.4	0.1
**Catheterization**	45%	0%	12.5%	21.2	**0.00002**
**Renal Disease (Overall)**	53.3%	25.0%	12.5%	16.99	**0.00020**
A] Acute Kidney Injury (AKI)	20.0%	7.1%	-	2.35	0.12
B] Chronic Kidney Disease (CKD)	20.0%	14.2%	12.5%	0.99	0.60
C] Nephrolithiasis (Calculi)	12.0%	7.1%	6.2%	0.91	0.63
D] Others (Lupus, Neurogenic bladder, Radiation cystitis, Hydroureteronephrosis (HUN)	8.0%	-	-	-	-

* The *p*-Value showing significance is marked in bold letters.

**Table 2:** Virulence gene carriage comparison in different levels of UTI.

Virulence Gene	Urosepsis Urine (n = 60)	Urosepsis Blood (n = 60)	Cystitis (n = 28)	Pyelonephritis (n = 32)	Chi-Square *X*^2^	*p*-Value *
Adhesin genes						
*papGII*	40 (66.7%)	39 (65.0%)	12 (42.9%)	18 (56.3%)	4.53	0.10
*papG III*	3 (5.0%)	2 (3.3%)	3 (10.7%)	(0%)	0.98	0.32
*papGI*	(0%)	(0%)	(0%)	(0%)	-	-
*papG IVa*	(0%)	(0%)	(0%)	(0%)	-	-
*papA*	41 (68.3%)	40 (66.7%)	13 (46.4%)	16(50.0%)	5.01	0.81
*papC*	43 (71.7%)	42 (70.0%)	13 (46.4%)	19(59.4%)	5.37	0.06
*afaC*	11 (18.3%)	11 (18.3%)	3 (10.7%)	3(9.4%)	1.73	0.41
*csgA*	58 (96.7%)	57 (95.0%)	28 (100.0%)	30 (93.8.%)	0.47	0.78
*fimH*	51 (85.0%)	50 (83.3%)	19 (67.9%)	26 (81.3%)	3.54	0.16
*sfaS*	4 (6.7%)	2 (3.3%)	3 (10.7%)	1 (3.1%)	1.38	0.50
*afa*/*Dr*	11 (18.3%)	11 (18.3%)	3 (10.7%)	5 (15.6%)	0.83	0.65
Toxin genes						
*cnf1*	20 (33.3%)	18 (30.0%)	4 (14.3%)	6 (18.8%)	4.60	0.10
*hlyA*	29 (48.3%)	24 (40.0%)	6 (21.4%)	10 (31.3%)	6.62	**0.03**
Iron uptake genes						
*iutA*	49 (81.7%)	49 (81.7%)	24 (85.7%)	29 (90.6%)	1.32	0.51
*iucD*	49 (81.7%)	49 (81.7%)	24 (85.7%)	29 (90.6%)	1.32	0.51

* The *p*-Value showing significance is marked in bold letters.

**Table 3:** The distribution of *papG* genes among the three levels of UTI.

	Distribution of *papG* Types Within the Infection Types				
Infection Type	GII (n = 68)	GII & GIII (n = 2)	GIII (n = 4)	Negative (n = 46)	Total
Cystitis	14.7% (n = 10)	100% (n = 2)	25% (n = 1)	33% (n = 15)	28
Pyelonephritis	26.5% (n = 18)	0% (n = 0)	0% (n = 0)	30% (n = 14)	32
Urosepsis	58.8% (n = 40)	0% (n = 0)	75% (n = 3)	37% (n = 17)	60
Total	68	2	4	46	120

**Table 4:** Distribution of virulence genes in relation to the *papG* genes carriage.

	*papGII* (n = 68)			*papG* Negative (n = 46) – Negative for *papGI*, *II*, *III*			*papGIII* (n = 4)			*papGII* and *papGIII* (n = 2)		
Virulence gene positive	Cystitis (n = 10)	PN * (n = 18)	Urosepsis (n = 40)	Cystitis (n = 15)	PN * (n = 14)	Urosepsis (n = 17)	Cystitis (n = 1)	PN * (n = 0)	Urosepsis (n = 3)	Cystitis (n = 2)	PN * (n = 0)	Urosepsis (n = 0)
*iutA*	100% (n = 10)	100% (n = 18)	95% (n = 38)	80% (n = 12)	78.6% (n = 11)	64.7% (n = 11)	0	0	0	100% (n = 2)	0	0
*afaC*	0	0	17.5% (n = 7)	20% (n = 3)	21.4% (n = 3)	23.5% (n = 4)	0	0	0	0	0	0
*papA*	100% (n = 10)	100% (n = 18)	97.5% (n = 39)	0	7.1% (n = 1)	0	100% (n = 1)	0	66.7% (n = 2)	100% (n = 2)	0	0
*papC*	100% (n = 10)	88.9% (n = 16)	100% (n = 40)	0	0	5.9% (n = 1)	100% (n = 1)	0	66.7% (n = 2)	100% (n = 2)	0	0
*cnf*	10% (n = 1)	33.3% (n = 6)	40% (n = 16)	0	0	11.7% (n = 2)	100% (n = 1)	0	66.7% (n = 2)	100% (n = 2)	0	0
*hlyA*	30% (n = 3)	50% (n = 9)	62.5% (n = 25)	0	7.1% (n = 1)	11.7% (n = 2)	100% (n = 1)	0	66.7% (n = 2)	100% (n = 2)	0	0
*fimH*	60% (n = 6)	77.8% (n = 14)	92.5% (n = 37)	80% (n = 12)	85.7% (n = 12)	76.5% (n = 13)	0	0	33.3% (n = 1)	50% (n = 1)	0	0
*sfa S*	20% (n = 2)	5.56% (n = 1)	2.5% (n = 1)	6.6% (n = 1)	0	0	0	0	100% (n = 3)	0	0	0
*csgA*	100% (n = 10)	88.9% (n = 16)	100% (n = 40)	100% (n = 15)	100% (n = 14)	88.2% (n = 15)	100% (n = 1)	0	100% (n = 3)	100% (n = 2)	0	0
*iucD*	100% (n = 10)	100% (n = 18)	95% (n = 38)	80% (n = 12)	78.6% (n = 11)	64.7% (n = 11)	0	0	0	100% (n = 2)	0	0
*afa/Dr*	0	5.56% (n = 1)	17.5% (n = 7)	20% (n = 3)	28.6% (n = 4)	23.5% (n = 4)	0	0	0	0	0	0
Renal disease	30% (n = 3)	22.2% (n = 4)	57.5% (n = 23)	26.7% (n = 4)	0	41.2% (n = 7)	0	0	66.7% (n = 2)	0	0	0
Catheterisation	0	11.1% (n = 2)	50% (n = 20)	0	14.3% (n = 2)	41.2% (n = 7)	0	0	33.3% (n = 1)	0	0	0

* PN—pyelonephritis.

## Data Availability

The raw data supporting the conclusions of this article will be made available by the authors on request.
